# Growth, Dietary Intake, and Vitamin D Receptor (VDR) Promoter Genotype in Indonesian School-Age Children

**DOI:** 10.3390/nu13092904

**Published:** 2021-08-24

**Authors:** Tiffany Cornelia Angelin, Saptawati Bardosono, Dewi Shinta, Umi Fahmida

**Affiliations:** 1 Southeast Asian Ministers of Education Organization Regional Center for Food and Nutrition (SEAMEO RECFON)-Pusat Kajian Gizi Regional, Universitas Indonesia, Jakarta 10430, Indonesia; tiffany.angelin@gmail.com (T.C.A.); dewishinta.deos@gmail.com (D.S.); 2 Department of Nutrition, Faculty of Medicine, Universitas Indonesia—Dr. Cipto Mangunkusumo General Hospital, Jakarta 10430, Indonesia; tati.bardo@yahoo.com

**Keywords:** stunting, children, height-for-age z-score (HAZ), dietary intake, VDR gene, Indonesia, vitamin D, calcium

## Abstract

Nutrition has been known as a predominant factor associated with stunting. However, some studies have discovered a genetic contribution in calcium absorption that will affect growth, known as the VDR gene. The aim of this study was to assess the association between VDR gene polymorphism and dietary intake towards height-for-age z-score (HAZ) of elementary school children in Malang District, East Java. This study analyzed the baseline of a randomized trial in East Java, Indonesia. School children aged 8–10 years old (*n* = 142) were included in this study. Energy, protein, calcium, and vitamin D intakes were obtained using 4-day 24-h dietary recalls. Two SNPs located in the promoter region of VDR gene were selected (rs11568820 and rs4516035) and analyzed using Real-Time PCR. The result showed a significant correlation between energy and protein intake with HAZ of the children (*p* = 0.030 and *p* = 0.016, respectively). The association between VDR gene and HAZ was not found (*p* > 0.05). Adjusted by other factors, protein intake was significantly correlated with HAZ (β = 0.034, 95% CI 0.015–0.052, *p* < 0.001, adj. R^2^ = 0.089). The children in our study had a favorable VDR gene genotype, however the effect of VDR gene promoter activity might not be revealed due to very low vitamin D and calcium intake to stimulate intestinal calcium absorption which in turn affects HAZ.

## 1. Introduction

In developing countries, childhood stunting remains as a major public health problem. The prevalence of stunting among children aged 5–12 years old in Indonesia was 23.6% and classified as a high public health problem [1,2]. Stunting has been related to many adverse consequences in the future life, as it negatively affects health, cognitive development, and economic aspect [3]. It has been known that the most predominant factor related to stunting was environmental factors such as nutrition and infectious diseases. Children with energy deficiency will experience growth retardation and loss of fat and muscle that might lead to increased morbidity and mortality [4]. Further evidence also suggests that energy deficit increases the need for protein and amino acids [5], which are required for the growth of children. Stunted children also have inadequate dietary protein intake and low serum amino acids as compared to their non-stunted counterparts [6]. In developing countries such as Indonesia, dietary protein intake is mainly obtained from plant-based sources, which are deficient in certain amino acids such as lysine and tryptophan [7]. Moreover, micronutrient intake, such as calcium and vitamin D also play an important role in growth of the children. Calcium is needed for skeletal calcium retention during growth, while vitamin D is needed for the body to absorb calcium and maintain serum calcium in a normal state to promote bone mineralization [8,9].

In recent years, some studies discovered a genetic contribution in calcium absorption that will affect growth of children, known as the Vitamin D Receptor (VDR) gene [10,11]. The interaction between calcitriol and VDR will induce the transcription of the gene that codes for calcium transporter channels [9]. Two SNPs (rs11568820 and rs4516035) located in the promoter region of the VDR gene have been known for their association with human height [12]. Promoter region is a regulatory region of DNA that contains specific DNA sequence recognized by transcription factors to regulate gene transcription. The first SNP of VDR gene resides in the binding site transcription factor Cdx2, while the second SNP resides in the binding site transcription factor GATA [12,13]. The presence of SNPs in the promoter region of a gene is expected to modify the identification and binding affinity of the promoter region to transcription factors, thereby may affect the amount of mRNA and VDR produced [14,15]. Some studies showed that T to C base substitution in both SNPs reduces transcriptional activity of VDR [12,13]. In general, both genetic and nutritional factors influence the growth of children. However, in Indonesia, the information about linear growth related to polymorphism of the VDR gene remains unclear. Thus, this study aims to assess the association between genetic factor (VDR gene polymorphism) and nutritional factors (macronutrient and micronutrient intake) towards height for age z-score (HAZ) of elementary school children in Malang District, East Java, as one of the district in Indonesia with high prevalence of stunting (20.6%) among school age children.

## 2. Materials and Methods

### 2.1. Study Design and Population

This study was a cross-sectional study, conducted as a part of bigger study by SEAMEO RECFON entitled “Association between Intakes of Protein, Calcium and Milk with Gene Expression and Linear Growth of School Aged Children” that has been registered at ClinicalTrials.gov as NCT03895151. Ethical approval was obtained from the Ethical Commission of Faculty of Medicine, Universitas Indonesia (No. 786/UN2.F1/ETIK/2018, with addendum No. ND−441/UN2.F1/ETIK/PPM.00.02/2019). Population of this study was elementary school children in Malang District, aged 8–10 years old, who agreed to participate in this study (informed consent signed by parents or guardians). The exclusion criteria of this study were students who are absent during blood collection, have a history of bleeding such as hemophilia, or have a disability. All subjects who fulfill the criteria were included in this analysis (*n* = 142). This sample size was calculated based on hypothesis test for difference in HAZ between two population means of VDR gene genotypes (0.38) [11] with standard deviation of 0.8, 0.05 significance level, and 80% power.

### 2.2. Dietary Intake Assessment

Dietary intake for energy, protein, calcium, and vitamin D were assessed using 24-h dietary recall with multiple-pass interviewing technique. This instrument was more suitable to be used on elementary school children than the semi quantitative-food frequency questionnaire (SQ-FFQ), as it was easier for them to remember the food they consumed in a previous day than the frequency of food they consumed in the last month. Dietary recall was conducted in 4 non-consecutive days. For vitamin D intake, a Food Composition database was developed from Vietnamese Food Composition Table (2007) [16] and United States Department of Agriculture (USDA) database of food composition (2019) [17] as resources to obtain the amount of vitamin D content in foods for the analyses. Energy under-reporting was estimated using Goldberg cut-off. Proportion at risk of inadequate calcium and vitamin D intakes were estimated using Estimated Average Requirement (EAR). Inadequate energy and protein intakes were defined when intakes of energy and protein were less than the requirement based on the actual body weight of each individual.

### 2.3. Sun Exposure Measurements

Sun exposure score of the participants was measured using sun exposure questionnaire. Student was asked to mention the amount of time spent for outdoor activities each day and which part of their body exposed to sunlight. The amount of time spent outdoors ≤5 min was scored 0, 5–30 min was scored 1, and ≥30 min was scored 2. For clothing or skin exposure while outdoors, there were four choices: face and hands only was (score = 1); face, hands and arms (score = 2); face, hands, and legs (score = 3); and almost all part of the body (score = 4).

The minimum daily sun exposure score was 0 and the maximum score was 8, which come from multiplication between scores for amount of time spent outdoor and score for skin exposed to sunlight. All seven days daily sun exposure scores were then summed to estimate the weekly sun exposure score with the minimum score was 0 and the maximum score was 56 [18].

### 2.4. Genotyping Procedure

Blood sample from 142 subjects was collected in EDTA vacutainer and buffy coat was isolated using centrifuge. DNA was isolated from buffy coat using QIAamp DNA Mini Kit. The isolated DNA was then mixed with TaqMan Universal GTXpressMastermix kits #4351379 which contain a pair of specific primer and probe mixture with ID C_2880805_10 for rs4516035 and ID C_2880808_10 for rs11568820. Total volume of the reaction was 10 μL which contained 2 μL template DNA (5 ng/μL), 5 μL TaqMan GTXpressMasterMix, 0.5 μL TaqMan SNP Genotyping Assay, and 2.5 μL Nuclease Free Water. The SNP Genotyping assay was analyzed using Real Time PCR system with 48-well thermos block instrument and StepOne V2.3 Software. A total of 40 cycles were performed. The cycling steps of the analysis were Hold (95 °C, 20 s), Denaturation (95 °C, 3 s), and Annealing Extension (60 °C, 20 s). The total running time was about one and a half hours for 40 cycles [19].

### 2.5. Statistical Analysis

Statistical analyses were performed using SPSS IBM 20.0 software. All variables were tested for its normal distribution using Kolmogorov–Smirnov Test. Genotyping results from PCR analysis of VDR gene polymorphism rs11568820 and rs4516035 were presented as a categorical data and descriptively analyzed to assess the frequency of different type of alleles and genotype distribution. To assess the correlation between dietary intake (energy, protein, calcium, and vitamin D) and HAZ, Pearson/Spearman test for correlation was used. The association between VDR gene polymorphism (rs11568820 and rs4516035) and HAZ was analyzed using T-Test/Mann–Whitney. Genetic and nutritional factors as determinant factors of HAZ were assessed together in multivariate analysis using multiple linear regression (enter and backward methods). Variables were included in multivariate analysis when the *p*-value < 0.25 in the binomial analyses with HAZ. Multiple linear regression analyses were conducted separately between SNP rs11568820 and rs4516035 due to existing multicollinearity, indicated by the interactions of those two SNPs as they lies on the same LD (linkage disequilibrium) block in VDR gene [20,21]. All statistical tests were considered significant at *p* < 0.05.

## 3. Results

A total of 142 children from six elementary schools (median age: 9 years) were included in this study. The mean HAZ was −0.993 (1.096) and the prevalence of stunting was 21.8%. Table 1 shows characteristics of the children.

Parents were mostly graduated from elementary and junior high schools. Most of the fathers worked as a laborer (53.5%), while mothers were mostly not working, i.e., housewives (56.3%). The most common infection experienced by the children in the past one month was respiratory infection, followed by diarrhea and helminths. In tropical areas, vitamin D intake is contributed from sunlight exposure. Sunlight exposure measured using the sun exposure questionnaire showed scores which ranged from 0 to 56 [18]. In this study, the median score for sun exposure was 33.5. It can be assumed that most of the children spend time outdoors for 5–30 min with almost all part of the body exposed, or spend time outdoors for more than 30 min with face, hands, and legs exposed.

Genotyping for the polymorphism of VDR gene showed that genotype distribution of rs11568820 was mostly heterozygote, while for rs4516035 were mostly wild homozygote (Table 2).

**Figure 1:**
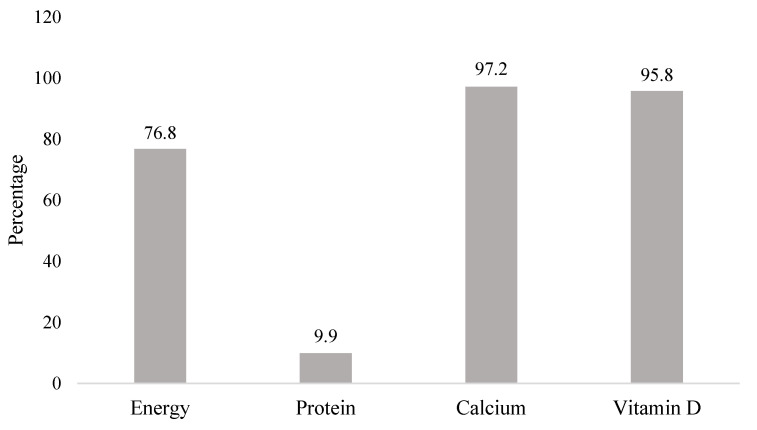
Proportions at risk on inadequate energy, protein, calcium, and vitamin D intakes amongst the school children.

Dietary intake of the children was adequate for protein, but inadequate for energy, calcium, and vitamin D intake. Energy and protein adequacy were calculated based on children’s body weight, while calcium and vitamin D adequacy were based on Estimated Average Requirement (EAR). Most of the children had inadequate micronutrient intake calcium and vitamin D; 97.2% and 95.8%, respectively (Figure 1). The median intake of calcium was 328.22 mg/day, while the median intake of vitamin D was 2.21 mcg/day which were far below the EAR (Table 3). Although 90.1% of children had adequate protein intake, there were 76.8% of children who had inadequate energy intake (Figure 1).

Bivariate analysis showed no association between VDR gene polymorphism with the HAZ of the children; *p* = 0.706 and *p* = 0.868 for rs11568820 and rs4516035, respectively (Table 4). On the other hand, Table 5 showed that energy and protein intake were significantly correlated with the HAZ of the children (*p* < 0.05).

Multivariate analysis was performed in two models. In the first model (Table 6), variables included were VDR gene, calcium intake, and vitamin D, in order to capture the role of those variables towards HAZ without adjusting for other factors. In the second model (Table 7), all variables were included. The result showed that protein intake was significantly correlated with HAZ (β = 0.034, 95% CI 0.015–0.052, *p* < 0.001, adj. R^2^ = 0.089).

Our study found that there was no significant difference between VDR gene polymorphism (rs11568820 and rs4516035) and height-for-age z-score (HAZ) of the children. HAZ was more significantly associated with protein intake.

Similar to our findings, another study also showed that rs4516035 was not significantly associated with body height [22]. Our finding differed from that found by Arai et al., who found that C allele of rs11568820 eliminates the Cdx2 binding site and reduces transcriptional activity of VDR to 70% of the T allele [13]. The increase in transcriptional activity in T allele of rs11568820 then increases intestinal calcium absorption and affects BMD, and it is likely related to body height [13]. Similar results, conducted by Jehan et al., showed that rs4516035 was associated with body height and serum calcium level [11]. T to C base substitution of rs4516035 may eliminate the GATA binding site and confers a lower VDR promoter activity [12]. The T allele of rs4516035 increases VDR transcriptional activity and it increases body height and serum calcium level [11]. The absence of association between vitamin D intake and HAZ in our study may be attributed to the fact that most of the children had adequate sunlight exposure (median score = 33.5 from maximum score of 56), and it is known that the major source of vitamin D is produced by exposure of skin to sunlight. Hanwell et al. stated that sun exposure score can be used to predict serum vitamin D concentration, accounting for 38% of the variability in serum vitamin D [18]. In addition, the low and narrow range of vitamin D intake amongst the children could have contributed to the lack of association between vitamin D intake and HAZ.

In our study, bivariate analysis showed that energy and protein intake were correlated with HAZ (*p* < 0.05). Tessema et al., found that stunted children had lower daily energy intake than non-stunted children [23]. Protein and essential amino acid are required for growth in children. Children at high risk of stunting may have limitations of essential amino acids in their diet, such as lysine and tryptophan [24]. A study in Filipino children showed that protein and calcium intakes were significantly associated with height of the children [25]. However, in our study, there was no correlation between calcium intake nor vitamin D intake and the HAZ score of the children. A previous study with 6–9-year-old Indian children also showed no correlation between growth and vitamin D status measured at 6–30 month of age [26]. The authors argued that growth of the children may be accounted more by deficiencies of other growth-limiting macro- and micronutrients such as calcium, zinc, and vitamin B12, and a lower proportion of animal source protein in food. In our study, stunted children had a lower intake of protein, fat, lysine (*p* < 0.05), zinc, and tryptophan (*p* < 0.1), but there was no difference between the two groups in calcium and vitamin D intakes. Although protein intake was adequate, only 43% of total protein originated from animal food sources. In addition, the narrow range of intakes of calcium and vitamin D in our study population may have also accounted for lack of association with HAZ.

Many factors affect linear growth which can lead to growth retardation. As well as genetic and nutritional factors, there were underlying factors and basic factors such as parental education and occupation, age, and gender. Multiple linear regression was performed to adjust those underlying and basic factors (*p* < 0.25). The analysis was performed for each SNPs of VDR gene and analyzed in two models. In first model, variables included were VDR gene, calcium intake, and vitamin D intake. The result (Table 6) showed that none of those factors were associated with HAZ of the children. Meanwhile, in second model (Table 7) calcium intake had a significant negative correlation with HAZ after protein intake was added. Calcium plays an important role in bone mineralization [27] and previous study has found that height was significantly related to calcium absorption [28]. However, while adequate vitamin D status is important to promote calcium absorption, several situations or conditions within gastrointestinal tract may directly inhibit intestinal calcium absorption. Protein is a nutrient which enhances calcium absorption, but high dietary protein intake could enhance urinary calcium excretion and is associated with hypercalciuria [9,29]. Several dietary components, such as caffeine, phytic acids, and oxalic acids which bind to calcium may also decrease calcium absorption [9]. Most of children in this study area commonly consume legumes, seeds, and beverages such as tea, coffee, and chocolate drink, which contain calcium inhibitors such as caffeine, phytic acid, and oxalic acid. The very low calcium intake (328.22 mg/day) and consumption of calcium inhibitor might therefore have contributed to the negative correlation with HAZ.

Our results showed that protein intake has a positive correlation with HAZ of the children (*p* < 0.001), in which every 1 g/day protein increases HAZ by 0.034. Inadequate dietary intake of protein and amino acids may adversely affect serum amino acid status, which may, in turn, reduce the growth of children [23]. The linear growth of children is dependent upon the chondral growth plate which is regulated by mTORC1 (the mechanistic target of rapamycin complex C1) and the availability of amino acids [30,31]. According to Laplante and Sabatini’s study, when amino acids are deficient in the diet, mTORC1 repress synthesis of both proteins and lipids, thus limiting cell growth [32]. Children at high risk of stunting may have limitations of essential amino acids in their diet, such as lysine and tryptophan [24].

In the multiple linear regression, we did not find the association between VDR gene polymorphism (rs11568820 and rs4516035) and HAZ of the children (*p* > 0.05). Jehan et al., observed that the association between VDR promoter genotype (rs4516035) and serum calcium levels was not found in 25(OH)D deficient children (<33 nmol/L) suggesting that a sufficient production of 1.25(OH)2D, the active form of vitamin D, is required to reveal the VDR promoter genotype effect [11]. The habitual consumption of low calcium diets is a major stimulus increasing the efficiency of intestinal calcium absorption through increased renal production of the active form of vitamin D (1.25[OH]2D) known as calcitriol [33]. Increased calcitriol stimulates calcium absorption in intestines by increasing calcium transporter channel through its genomic mechanism. With respect to genomic effects on intestinal calcium absorption, calcitriol is transported into the enterocyte and carried into the nucleus, where it interacts with nuclear VDRs to directly regulate specific genes encoding for proteins involved in calcium uptake and transport, such as calbindin D9k, calcium channel transporter, Ca^2+^ ATPase, and claudin protein [9]. Calcium absorption in the gut is dependent upon adequate vitamin D status, and a previous study had shown that the low 25(OH)D concentration was insufficient for the formation of enough 1.25(OH)2D needed to increase intestinal calcium absorption [34]. The limitation of our study is that vitamin D status was not measured. To find out whether the VDR genotype affects height, further research is needed to assess vitamin D levels as a primary indicator, to see the role of VDR in the calcium absorption process; this might affect height of the children. It is likely that, as the children in this study area had very low vitamin D intake, their vitamin D levels might be low, so the effect of VDR promoter activity was not revealed. For further study, we recommend the assessment of calcium and vitamin D concentrations, as well as other biochemical analyses related to height.

## 4. Conclusions

Protein intake statistically showed that it has a direct influence towards HAZ. Every 1 g/day protein increase HAZ by 0.034. Genotype analysis also showed that Indonesian children had a favorable VDR gene genotype, however the effect of VDR gene promoter activity on HAZ might not be revealed due to very low vitamin D intake to stimulate intestinal calcium absorption, which in turn affects the HAZ of the children.

## Figures and Tables

**Table 1:** Characteristics of the children (*n* = 142).

Factors	*n* (%)
Age (year) *	9 (8–10)
Sex	
Boy	85 (59.9)
Mother Education Level	
≤9 years	100 (70.4)
> 9 years	42 (29.6)
Father Education Level	
≤9 years	98 (69)
>9 years	44 (31)
Father Occupation	
Government employee	3 (2.1)
Private employee	20 (14.1)
Trader	41 (28.9)
Laborer	76 (53.5)
Not working	2 (1.4)
Mother Occupation	
Working	62 (43.7)
Not Working	80 (56.3)
History of Infectious Diseases	
a. Diarrhea b. Malaria c. Helminths Respiratory Infection	21 (14.8) 0 (0) 3 (2.1) 64 (45.1)
Sun exposure score (7 days) *	33.5 (2–56)

* The data was presented as median (min–max).

**Table 2:** Variant allele frequency and genotype distribution of VDR gene polymorphism amongst the school children (*n* = 142).

SNPs	Variant T Allele Frequency (%)	Wild-Type Homozygote (TT) *n* (%)	Heterozygote (CT) *n* (%)	Mutant Homozygote (CC) *n* (%)
rs11568820	40.85	27 (19)	62 (43.7)	53 (37.3)
rs4516035	95.42	129 (90.8)	13 (9.2)	0 (0)

**Table 3:** Dietary intake of the school children (*n* = 142).

Nutrient	Mean ± SD Median (min–max)	Indonesian RDA (AKG, 2019)	EAR
Energy (Kcal)	1390 ± 355	1650 *	N/A
Protein (g/day)	39 (19–111)	40 *	N/A
Calcium (mg/day)	328.22 (118.68–1010.55)	1000	800
Vitamin D (mcg/day)	2.21 (0.09–17.77)	15	10

* Based on median body weight used in AKG (27 kg); actual median body weight in this study population was 25 kg; N/A: not applicable.

**Table 4:** Association between VDR gene polymorphism and HAZ.

SNPs	*n*	Height-for-Age Z-Score (HAZ)	
		Mean ± SD	*p* Value ^a^
rs4516035 ^b^			0.868
C	13	−1.04 ± 0.87	
T	129	−0.99 ± 1.12	
rs11568820 ^b^			0.706
C	115	−1.01 ± 1.12	
T	27	−0.92 ± 1.02	

^a^ Using Independent T-test; ^b^ C = CT and CC genotype; and T = TT genotype.

**Table 5:** Correlation between dietary intake and HAZ (*n* = 142).

Nutrient Intake	Height-for-Age Z-Score (HAZ)	
	r	*p* Value
Energy (kcal/day)	0.183	0.030 ^a^
Protein (g/day)	0.203	0.016 ^b^
Calcium (mg/day)	0.021	0.804 ^b^
Vitamin D (mcg/day)	0.018	0.829 ^b^

^a^ Using Pearson correlation; ^b^ Using Spearman correlation.

**Table 6:** Multivariate analysis between HAZ, VDR gene, calcium and vitamin D intakes (Model 1).

Variables ^1^	rs11568820								rs4516035							
	Full Model ^a^				Final Model ^b^				Full Model ^a^				Final Model ^b^			
	B	95% CI	*p* Value	Adj. R^2^	B	95% CI	*p* Value	Adj. R^2^	B	95% CI	*p* Value	Adj. R^2^	B	95% CI	*p* Value	Adj. R^2^
(Constant)	−0.951	−1.386–(−0.516)	< 0.001	0.003	−0.949	−1.208–(−0.691)	< 0.001	0.002	−1.000	−1.720–(−0.279)	0.007	0.002	−0.949	−1.208–(−0.691)	< 0.001	0.002
VDR gene ^2^	0.109	−0.365–0.583	0.650		N/A	N/A	N/A		0.064	−0.575–0.703	0.844		N/A	N/A	N/A	
Calcium Intake (mg/day)	0.000	−0.001–0.001	0.942		N/A	N/A	N/A		0.000	−0.001–0.001	0.972		N/A	N/A	N/A	
Vitamin D Intake (mcg/day)	−0.015	−0.081–0.052	0.666		−0.014	−0.072–0.044	0.641		−0.014	−0.080–0.053	0.687		−0.014	−0.072–0.044	0.641	

^1^ N = 142; ^a^ Linear regression using enter method; ^b^ Linear regression using backward method; *p*-value ANOVA = 0.001; ^2^ rs11568820 and rs4516035: CC and CT (0), TT (1); Dependent Variable: Height for Age Z-score (HAZ); and N/A: not applicable.

**Table 7:** Multivariate analysis between HAZ, VDR gene, calcium and vitamin D intakes and other potential factors (Model 2).

Variables ^1^	rs11568820								rs4516035							
	Full Model ^a^				Final Model ^b^				Full Model ^a^				Final Model ^b^			
	B	95% CI	*p* Value	Adj. R^2^	B	95% CI	*p* Value	Adj. R^2^	B	95% CI	*p* Value	Adj. R^2^	B	95% CI	*p* Value	Adj. R^2^
(Constant)	−1.903	−2.742–(−1.064)	< 0.001	0.061	−1.811	−2.403–(−1.220)	< 0.001	0.089	−1.783	−2.764–(−0.802)	< 0.001	0.059	−1.811	−2.403–(−1.220)	< 0.001	0.089
VDR gene ^2^	0.156	−0.308–0.621	0.507		N/A	N/A	N/A		−0.095	−0.725–0.535	0.766		N/A	N/A	N/A	
Energy Intake (kcal/day)	0.000	−0.001–0.001	0.833		N/A	N/A	N/A		0.000	−0.001–0.001	0.923		N/A	N/A	N/A	
Protein Intake (g/day)	0.032	0.004–0.060	0.027 *		0.034	0.015–0.052	<0.001 *		0.033	0.004–0.061	0.024 *		0.034	0.015–0.052	<0.001 *	
Calcium Intake (mg/day)	−0.002	−0.003–0.000	0.022 *		−0.002	−0.003–0.000	0.013 *		−0.002	−0.003–0.000	0.026 *		−0.002	−0.003–0.000	0.013 *	
Vitamin D Intake (mcg/day)	0.005	−0.060–0.070	0.879		N/A	N/A	N/A		0.008	−0.058– 0.073	0.811		N/A	N/A	N/A	
Sun Exposure Score	0.000	−0.013–0.013	0.962		N/A	N/A	N/A		0.000	−0.013–0.013	0.976		N/A	N/A	N/A	
Father Education ^3^	0.243	−0.213–0.699	0.294		0.327	−0.054–0.708	0.092		0.268	−0.193–0.729	0.252		0.327	−0.054–0.708	0.092	
Mother Education ^3^	0.149	−0.304–0.602	0.516		N/A	N/A	N/A		0.149	−0.304–0.602	0.517		N/A	N/A	N/A	

^1^ N = 142; ^a^ Linear regression using enter method; ^b^ Linear regression using backward method; *p*-value ANOVA = 0.001; ^2^ rs11568820 and rs4516035: CC and CT (0), TT (1); ^3^ Education Levels: ≤9 years (0), >9 years (1); Dependent Variable: Height for Age Z-score (HAZ); * significantly correlated (*p* < 0.05); and N/A: not applicable.

## Data Availability

The data presented in this study are available on request from the corresponding author. The data are not publicly available due to it being part of the main study which has not been published.
